# Discovery of Inhibitors of Insulin-Regulated Aminopeptidase as Cognitive Enhancers

**DOI:** 10.1155/2012/789671

**Published:** 2012-12-04

**Authors:** Hanna Andersson, Mathias Hallberg

**Affiliations:** ^1^Division of Organic Pharmaceutical Chemistry, Department of Medicinal Chemistry, BMC, Uppsala University, P.O. Box 574, 751 23 Uppsala, Sweden; ^2^Division of Biological Research on Drug Dependence, Department of Pharmaceutical Biosciences, BMC, Uppsala University, P.O. Box 591, 751 24 Uppsala, Sweden

## Abstract

The hexapeptide angiotensin IV (Ang IV) is a metabolite of angiotensin II (Ang II) and plays a central role in the brain. It was reported more than two decades ago that intracerebroventricular injection of Ang IV improved memory and learning in the rat. Several hypotheses have been put forward to explain the positive effects of Ang IV and related analogues on cognition. It has been proposed that the insulin-regulated aminopeptidase (IRAP) is the main target of Ang IV. This paper discusses progress in the discovery of inhibitors of IRAP as potential enhancers of cognitive functions. Very potent inhibitors of the protease have been synthesised, but pharmacokinetic issues (including problems associated with crossing the blood-brain barrier) remain to be solved. The paper also briefly presents an overview of the status in the discovery of inhibitors of ACE and renin, and of AT1R antagonists and AT2R agonists, in order to enable other discovery processes within the RAS system to be compared. The paper focuses on the relationship between binding affinities/inhibition capacity and the structures of the ligands that interact with the target proteins.

## 1. Introduction

Neuropeptides participate in the transmission or modulation of signals in the central nervous system (CNS) [1]. Hence, these peptides are engaged in neurological functions that include those related to cognition and memory, mood, the experience of pain, stress, reaction to reward, control of the intake of food, and neuroendocrinological regulation. The physiological action of neuropeptides is terminated by proteolytic degradation, and this is most often mediated by extracellular proteases anchored in the cell membrane. In this respect, neuropeptides differ from classic transmitters. Limited hydrolysis of neuroactive peptides may lead to the fragments being formed with either similar or very different biological activities [2]. The conversion of angiotensin II (Ang II) to angiotensin IV (Ang IV) is a good example of the latter. This type of biotransformation results from the action of more or less specific endoproteases. Several proteases that are capable of releasing bioactive fragments from their substrates have been identified in various CNS tissues [3, 4].

We discuss in this paper the renin-angiotensin system (RAS) and describe briefly how the two proteases, the angiotensin converting enzyme (ACE) and renin, have served and continue to serve as drug targets. We discuss briefly the two major receptors of the parent peptide angiotensin II, AT1R and AT2R, and we describe related antagonists and agonists to these receptors. Finally, we direct our focus to the hexapeptide Ang IV, which plays a central role in the brain. It has been suggested that the insulin-regulated aminopeptidase (IRAP) is the major target for Ang IV in the brain, and we therefore discuss in more detail recent progress in the discovery of inhibitors of IRAP. This paper concentrates on the molecular structures of the ligands that interact with the target proteins.

## 2. Proteolytic Processing

**Figure 1:**
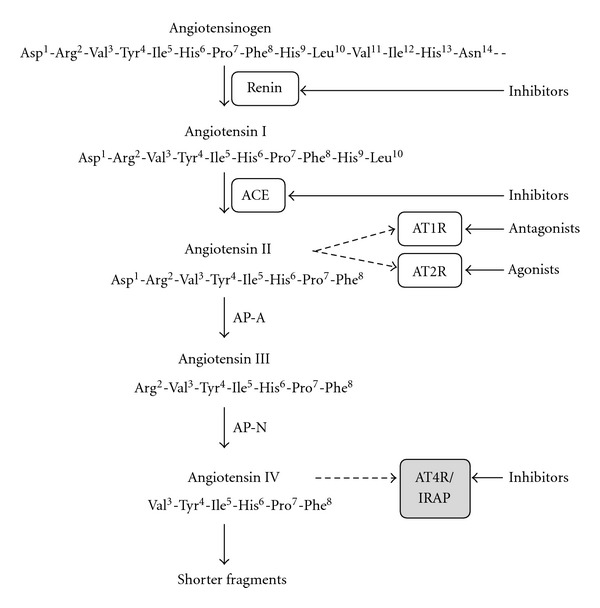
A part of the renin-angiotensin system (RAS), including selected degradation products and drug targets.

Angiotensin II (Ang II) is formed from angiotensin I (Ang I), which is an essentially inactive peptide derived from circulating and tissue angiotensinogen (Figure 1). The aspartyl protease renin liberates Ang I from angiotensinogen. The proteolytic cleavage of angiotensin I to produce Ang II is mediated mainly by the metalloproteinase ACE, an established target for drug therapy. Enzymatic cleavage by chymase, carboxypeptidase, catepsin G or tonin are alternative routes by which Ang II can be produced [5]. As in the cases of the tachykinins and the opioid peptides, metabolism of Ang II results in the formation of several fragments with biological activities that differ from those of the parent peptides. Proteolytic cleavage by glutamyl aminopeptidase A (AP-A) and membrane alanyl aminopeptidase N (AP-N), for example, results in the sequential removal of single amino acid residues from the N-terminal end of the peptide, to form Ang III (Ang II(2–8)) and Ang IV (Ang II(3–8)), respectively [6]. These peptides are important neuropeptide fragments in the CNS [7–10]. Ang IV plays a particularly important role, and its mechanism of action is distinct [11–14]. It is noteworthy that Ang IV can be formed by the action of aminopeptidases on Ang I before it is converted to Ang II [15]. A previously unknown human Ang II-related peptide, denoted Ang A, has recently been discovered [16]. This peptide, (Ala^1^)-Ang II, is not a product of proteolysis but is derived from decarboxylation of the aspartic acid residue of Ang II [16]. It acts as a full agonist with properties that are similar to those of Ang II [17].

Chymotrypsin and dipeptidyl carboxypeptidase can further process Ang IV and the fragment Ang (3–7) to form inactive fragments and amino acid residues [18–23]. Ang (3–7) is formed from Ang IV by carboxypeptidase P (Carb-P) and propyl oligopeptidase (PO) cleavage. Chymotrypsin can hydrolyse bonds to Val, Tyr, and Ile, and this is an important property to consider when designing metabolically stable Ang IV analogues and Ang IV peptide mimetics. Furthermore, Ang II can be converted to the bioactive Ang (1–7) by the proteolytic removal of the C-terminal phenylalanine by Carb-P [12], the action of the monopeptidase ACE2 [24], and by ACE cleavage of the Phe-His from Ang (1–9) [25].

Figure 1 shows selected degradation products and five major drug targets. There are several other potential targets for drugs in the RAS, such as the Ang (1–7)/Mas receptor [26, 27] and the aminopeptidase A (AP-A), but these will not be discussed here (for a recent paper, see [28]). Neither will antagonists to AT2R as potential future drugs be discussed. This review will focus on the discovery of IRAP inhibitors.

## 3. Inhibitors of Angiotensin Converting Enzyme and Renin

Angiotensin II (Asp-Arg-Val-Tyr-Ile-His-Pro-Phe) is an important modulator of cardiovascular function and exerts a pronounced hypertensive effect. Forty years ago, it was discovered that minor modifications of the amino acid residue sequence of Ang II, such as the replacement of the phenylalanine residue at the C-terminal by a residue with an aliphatic side chain, created peptides that block the action of Ang II. Two such peptides, saralasin ((Sar^1^, Ala^8^)-Ang II) and sarile ((Sar^1^, Ile^8^)-Ang II), in which the N-terminal sarcosine residue enhances the effect, were evaluated in clinical trials [29, 30]. However, due to the peptidic character neither saralasin, approved by FDA for limited applications, nor the more potent sarile found long term use in clinic [31–33]. They did, however, become important research tools and clinical observations from treatment with these two peptides confirmed that the RAS is a very relevant target for drug intervention. Hence, the two major proteases, renin and ACE, that are responsible for the degradation of the precursor protein angiotensinogen to the effector peptide Ang II, became attractive drug targets (Figure 1).

**Figure 2:**
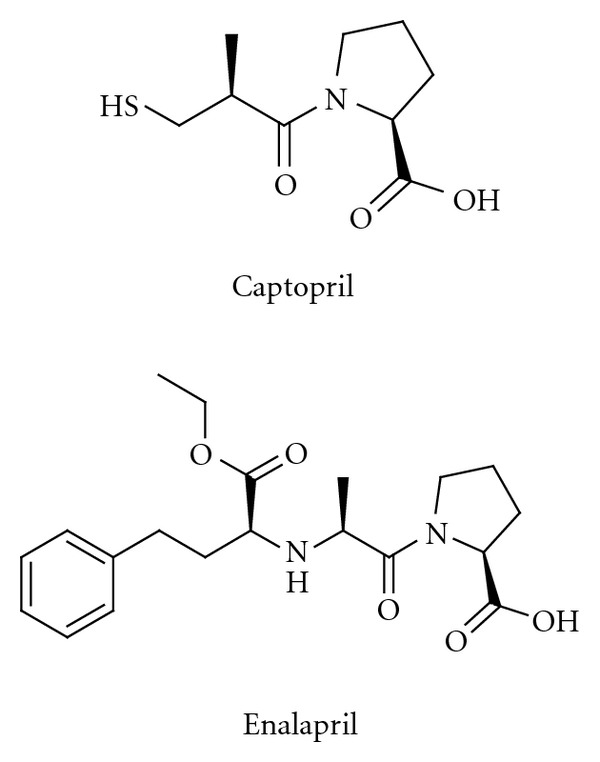
Captopril and enalapril, inhibitors of the angiotensin converting enzyme (ACE).

ACE inhibitors were subsequently disclosed [34, 35]. The design of the first ACE inhibitor took advantage of the similarity of ACE to the metalloproteinase carboxypeptidase A, which is inhibited by 2-benzyl succinic acid. It was also known that isolated peptides from extracts of venom from the Brazilian pit viper *Bothrops jararaca* have an antihypertensive effect, inhibiting the conversion of Ang I to Ang II. These two insights allowed the elegant and very rapid discovery of the zinc-binding thiol compound captopril, which entered the market in 1978. The nanopeptide (Pyr-Trp-Pro-Arg-Pro-Gln-Ile-Pro-Pro), known as “teprotide,” had the highest *in vivo* potency among the venom peptides. It contains a proline residue at the C-terminal that is retained in captopril (Figure 2) [36]. Attempts to improve metabolic stability and to minimize the principal side effects of captopril, rashes and loss of taste, that it was hypothesized that the thiol group was responsible for, led to the once daily prodrug enalapril (Figure 2) that entered the market in 1985 [37, 38]. In enalapril, the thiol group had been replaced by a zinc-coordinating carboxyl group. The high-resolution X-ray crystal structure of enalapril (after liberation of the free carboxyl group from the prodrug) is now available [39] and shows that the N-terminal carboxyl group interacts with the catalytic zinc ion at the active site of ACE. The 3D structure of the membrane-bound ACE, however, was not known when the first ACE inhibitors were designed. Subsequently there was a large number of ACE inhibitors introduced, for example, ramipril, quinapril, perindopril, lisinopril, and benazepril, that today are extensively used in clinic [40].

**Figure 3:**
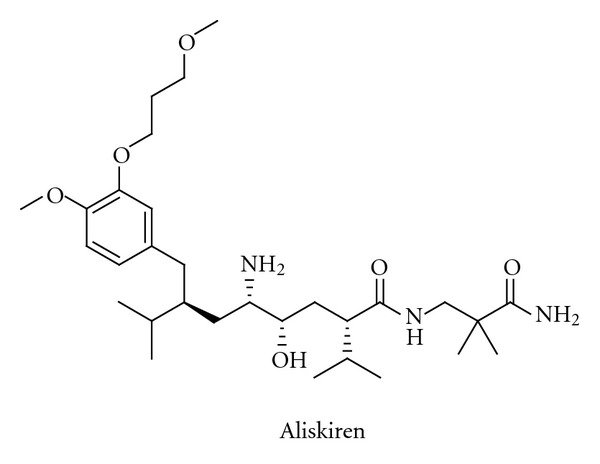
The structure of the renin inhibitor aliskiren.

Attempts to inhibit the other major protease involved in the proteolytic processing, the aspartyl protease renin, continued for decades, but it was not until 2007 that the first such inhibitor, aliskiren (Figure 3), reached the market [41–43]. The development of renin inhibitors was often hampered by the peptidic character of the potential inhibitors being studied, leading as it did to limited metabolic stability, poor absorption, and, as a consequence of this, low oral bioavailability. It was, furthermore, difficult to predict effects in humans from results obtained in animal models. The compounds were transition-state analogues, in which the peptide bond to be cleaved had been substituted by a group that mimicked the transition state. The design of the transition-state analogue aliskiren was aided by modelling and the availability of X-ray structures [44]. Experience gained during the renin inhibitor programmes benefited greatly programmes to discover HIV protease inhibitors, which were, in fact, on the market ten years before aliskiren. Significant progress has recently been made in identifying new potent non-peptide “direct renin inhibitors”, but no such inhibitors have progressed to late stage development.

## 4. Angiotensin II AT1 Receptor Antagonists and AT2 Receptor Agonists

**Figure 4:**
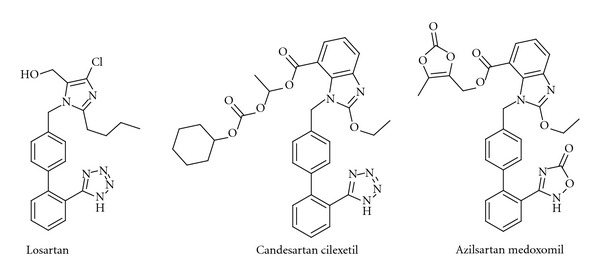
Three examples of Ang II AT1 receptor blockers (ARBs).

The first angiotensin receptor blocker (ARB) to be used in the clinic, the peptide saralasin, was not orally active and its duration of action was very short. Clinical results obtained with saralasin, however, demonstrated clearly that the Ang II receptor was a suitable target for drugs. The first non-peptide ARB, losartan (Figure 4), was introduced onto the market in 1995. Losartan is characterized by the tetrazole moiety, which serves as a carboxylate bioisostere, at the biphenyl unit and by the readily oxidized hydroxymethyl group. Losartan is converted *in vivo* by oxidation of the hydroxymethyl group to the more potent carboxylic acid metabolite. For a review on the discovery of angiotensin receptor blockers, see [45]. Several other AT1R antagonists are now in clinical use, for example, eprosartan, olmesartan, telmisartan, valsartan, and candesartan. The latter is administered as a prodrug, candesartan cilexetil (Figure 4). This liberates candesartan, which binds strongly to the AT1 receptor [46, 47]. It is believed that an optimal clinical effect of these drugs, known collectively as “sartans,” requires high levels of target occupancy [48, 49]. It should be mentioned in this context that an increasing number of examples in the literature suggest that the *in vivo* duration of drug action of AT1R blockers, for example, depends not only on macroscopic pharmacokinetic properties such as plasma half-life and the time needed to equilibrate between the plasma and the effect compartments, but also on long-lasting target binding and rebinding [50–52]. The prodrug azilsartan medoxomil (Figure 4), which has a less common carboxylic acid bioisostere attached to the biphenyl scaffold, was approved in 2011 and is the latest member of the ARB group [53–55]. It is well established that AT1R antagonists are at least as effective as ACE inhibitors, *β*-blockers, and calcium channel antagonists in reducing cardiovascular morbidity and mortality [56, 57]. Furthermore, it is reported that losartan [58] and candesartan [59–61] have positive effects on cognition in elderly patients. Interestingly, TRV120023 representing a *β*-arrestin-biased AT1R ligand has cardioprotective and functional properties *in vivo* which are distinct from losartan. It has been suggest that this novel class of drugs that is G protein independent but *β*-arrestin selective may provide an advantage over conventional ARBs by supporting cardiac function and reducing cellular injury during acute cardiac injury [62, 63]. Anyhow, efforts to develop new and better chemical entities that block the AT1 receptor are limited. There is strong competition in this research field, which may partly explain this decision, but efforts to develop single compounds that can block both the AT1R and the receptor of the very potent vasoconstrictor endothelin A are in progress [64]. Furthermore, dual antagonism of AT1R and neutral endopeptidase inhibition has recently attracted interest. Clinical results from the dual inhibitor LCZ696, now in phases II-III, are promising [65].

Recently, the AT2 receptor has emerged as a new target for drug therapy [66–68]. The AT2 receptor is abundant in fetal tissues but in adults this G protein-coupled receptor remains abundant only in certain tissues such as vascular endothelium [69] and brain areas [70, 71]. The AT2 receptor is present in higher density in distinct regions of the brain and it has been suggested that it is involved in growth development and exploratory behaviour [72, 73]. It is expressed in the locus coeruleus, ventral and dorsal parts of lateral septum, the superior colliculus, and subthalamic nucleus, in the nuclei of many cells of the thalamus, and in nuclei in cells of the inferior olive. Both the AT1 and AT2 receptors are expressed in the cingulate cortex, the molecular layer of the cerebellar cortex, the superior colliculus and paraventricular nuclei [70, 74–76]. AT2R RNA and the receptor protein have recently been identified in the substantia nigra pars compacta [77] and in the hippocampus [78, 79]. Thus, the receptor is present in the adult in areas associated with control and learning of motor activity, sensory areas, and selected structures of the limbic system [80]. It has been suggested that modulation of AT2R signalling can improve cognitive performance in persons with Alzheimer's disease (AD) not only through the action of an AT2R agonist on blood flow/brain microcirculation but also through its more specific effects on neurons [81]. Activation of the AT2 receptor affects neuronal cell differentiation and nerve regeneration [82–84]. Interestingly, several peripheral effects that are mediated through the AT2 receptor oppose other effects that are mediated through the AT1 receptor, suggesting that a similar balance may exist in the CNS [85, 86]. It is worth noting that the AT2 receptor is re-expressed in some disease conditions such as heart failure, renal failure, myocardial infarction, hypertension, and some brain disorders [71, 87–92]. The AT2R mediates vasodilatory, antiproliferative and anti-inflammatory effects [93].

**Figure 5:**
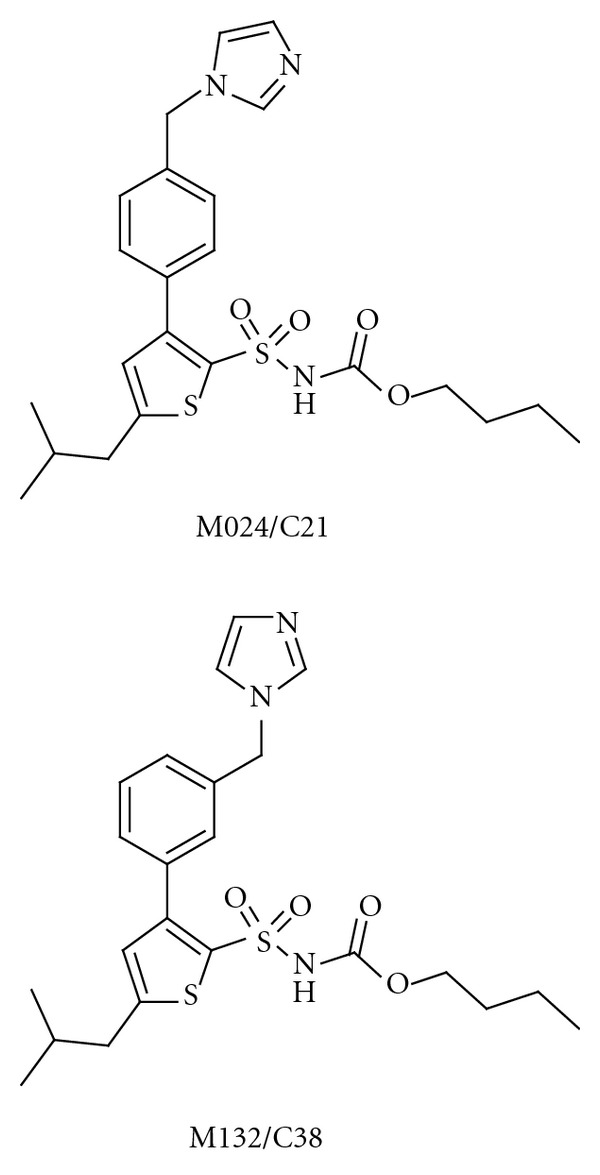
The structures of the selective AT2 receptor agonist M024/C21 and the structurally similar M132/C38, which acts as an AT2 receptor antagonist.

Several potent drug-like and selective AT2R agonists have been disclosed [94–96]. The first of these receptor-selective agonists, M024 or “compound 21” (M024/C21, Figure 5), was first reported in 2004, and has been extensively studied since then [94]. It possesses a sulfonyl carbamate entity as a metabolically stable carboxylic acid isostere and exhibits a striking structural similarity to several AT1R antagonists. Compound M024/C21, developed through a series of modifications to the non-selective AT1R agonist L-162,313 [97], stimulates neurite outgrowth in neuronal cells (which express only AT2R) [94] through the sustained activation of p42/p44 mapk. It decreases dopamine synthesis in the rat striatum, and some results suggest that the AT1 and AT2 receptors in the striatum exert opposite effects on dopamine synthesis, rather than dopamine release [98]. The compound enhances cognitive functions in mice [81, 99]. It improves myocardial function independently of blood pressure after myocardial infarction in normotensive Wistar rats [100] and has a pronounced anti-inflammatory effect [101]. M024/C21 gives improved vascular stiffness and lower collagen concentrations in the aorta and myocardium of stroke-prone spontaneously hypertensive rats [102]. It was recently suggested that the combination of M024/C21 with antihypertensive treatment might lead to vasculoprotective effects even beyond the blood-pressure-reducing effect [66, 103].

It is expected that bioavailability in the brain of the drug-like M024/C21 will be low [104]. This is expected to be the case also for M132/C38 (Figure 5), a selective AT2R antagonist with a very similar structure as M024/C21. Nevertheless, this AT2R agonist/antagonist pair, in which the two compounds possess similar pharmacokinetics, should be an important and useful tool in studying the RAS. Furthermore, these molecules may serve as a starting point in medicinal chemistry programmes aimed at discovering molecules that are active as AT2R agonists in the brain after oral administration. No selective AT2R agonists have still entered phase I clinical trial. On the contrary, EMA401, a lipophilic structural analogue of the commonly used research tool, the AT2 receptor antagonist PD123319 is in clinical trials. This antagonist is developed for neuropathic pain [105].

## 5. Inhibitors of Insulin-Regulated Aminopeptidase

**Figure 6:**
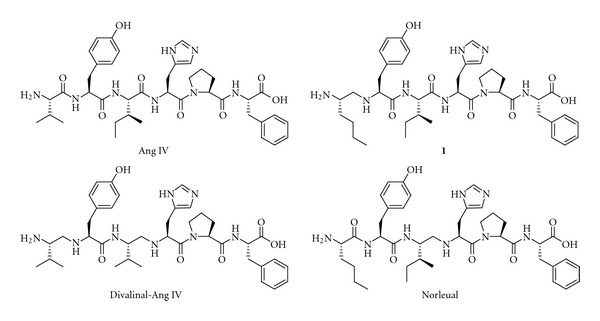
Ang IV and three hexapeptide analogues that incorporate one or two reduced peptide bonds (Ψ[CH_2_NH]) as peptide bond bioisostere.

In 1988, Braszko et al. reported that intracerebroventricular (i.c.v.) injection of the Ang II metabolite Ang IV (Val-Tyr-Ile-His-Pro-Phe, Figure 6) improved memory and learning in the rat [106]. They showed that Ang IV affects motor activity, the performance of passive avoidance, and a conditioned avoidance response. Various animal models were subsequently investigated, and results were obtained for Barnes maze, swim mazes, and radial arm mazes [8, 14, 107–110]. Not only Ang IV but also related analogues were studied, such as the endogenous LVV-hemorphin-7 (Leu-Val-Val-Tyr-Pro-Trp-Thr-Glu-Arg-Phe), which has structural similarities to Ang IV at the N-terminal part of the peptide, in that it has a tyrosine residue attached to lipophilic amino acid residues [111]. LVV-hemorphin-7 is a powerful promoter of memory retention and retrieval in rats [107]. The observation that Ang IV improves processes related to memory and learning has attracted considerable interest in recent years. Excellent reviews have been published describing the role of Ang IV in the brain [112–114]. Efficient new chemical entities for the treatment of the cognitive decline associated with Alzheimer's disease, brain trauma, and cerebral ischemia are needed since the clinical studies of the cholinesterase inhibitors and NMDA antagonists used today have been mostly disappointing [115–118]. Thus, new improved enhancers of cognitive functions are desired and the receptor(s) involved in the beneficial effects of Ang IV has emerged as a relevant new target for drug intervention.

A specific binding site for Ang IV was identified in 1992, and was later named the AT4 receptor [119–121]. Harding et al. found high densities of binding sites in areas of the brain associated with cognitive, sensory and motor functions, including the hippocampus [120].

### 5.1. Ligands with Affinity to the AT4 Receptor

Shortly after the discovery of the binding sites, systematic structure-activity relationship studies (SAR) were commenced by Wright and Harding and it became clear that the Val-Tyr-Ile tripeptide motif in the N-terminal part of Ang IV was of critical importance for binding affinity [122]. This conclusion relied on classic glycine and d-amino acid scans and various other alterations of the amino acid residues of Ang IV [123, 124]. Hydrophobic residues at position one, and norleucine in particular, rendered very high binding affinities, while substitution of the N-terminal amine by acetylation or methylation lead to low affinity ligands. Thus, a residue with straight aliphatic side-chain combined with a primary amine function was found to be preferred in the N-terminal. The C-terminal part, on the other hand, could be altered without affecting the binding affinity dramatically. However, truncations with the exception of removal of the C-terminal phenylalanine residue were found to be unproductive [122].

The hexapeptide Ang IV and its peptide analogues are prone to undergo proteolytic cleavage but a significant improvement of the metabolic stability could be achieved by reduction of the peptide bond between Val^1^ and Tyr^2^ of Ang IV with most of the affinity retained. Analogue **1** (Figure 6) constitute another such example, encompassing a reduced peptide bond (Ψ[CH_2_NH]) between residues one and two and thus two amine sites that can be protonated [123]. Divalinal-Ang IV and norleual (Figure 6) are two other pseudopeptides comprising reduced peptide bonds that were studied in some detail and served as important research tools [125, 126].

**Figure 7:**
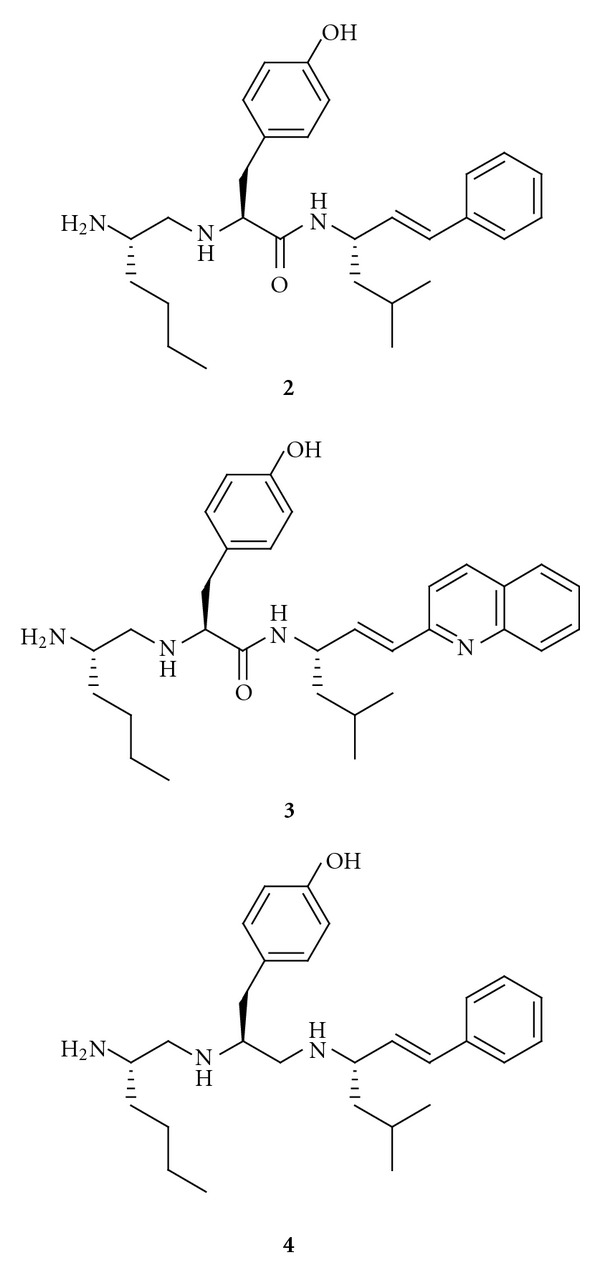
Highaffinity Ang IV receptor binding analogues that incorporate one or two reduced peptide bonds (Ψ[CH_2_NH]) and a styrene moiety replacing the C-terminal tripeptide His-Pro-Phe of Ang IV.

Kobori et al. at Taisho Pharmaceuticals filed patent applications in the late 1990s that disclosed a series of compounds that bind strongly to guinea pig hippocampus membranes [127, 128]. They had deduced this from competitive experiments with radiolabeled [^125^I]Ang IV. The compounds with the highest binding affinity are characterized by a straight fourcarbon chain at position one and a reduced amide bond (Ψ[CH_2_NH]) between residues one and two. These high-affinity compounds have also a styrene moiety that replaces His-Pro-Phe in Ang IV, (compound **2** and quinoline **3** in Figure 7) and they have IC50 values lower than 1 nM. Ligand **4** in Figure 7 has three basic amino groups and was produced by reducing the amide bond between residues two and three in compound **2**. This compound has a binding affinity for hippocampus membranes that is 40 times lower than that of compound **2**. A ligand essentially devoid of affinity was obtained by maintaining the peptide bond between residues one and two intact and reducing the bond (Ψ[CH_2_NH]) between residues two and three. Furthermore, reduction of the *trans* double bond at the C-terminal leads to considerably lower affinities. The potent ligands synthesized by Kobori et al. have to the best of our knowledge not been evaluated as IRAP inhibitors.

### 5.2. Inhibitors of Insulin-Regulated Aminopeptidase (IRAP)

A receptor for Ang IV was purified from bovine adrenal membranes in 2001. This AT4 receptor was identified as the insulin-regulated aminopeptidase (IRAP) [129]. The IRAP/AT_4_ receptor has attracted considerable interest in recent years as a potential target for pharmaceuticals aimed for the treatment of cognitive disorders [112, 130–133]. IRAP is a single-spanning transmembrane zinc-metallopeptidase that belongs to the M1 family of aminopeptidases. IRAP has been identified as cystinyl aminopeptidase (CAP, EC 3.4.11.3), placental leucine aminopeptidase (P-LAP, soluble human homologue), oxytocinase, gp160, or vp165 [134–137]. The insulin-regulated aminopeptidase has been cloned and characterized in adipocytes in vesicles that contain the insulin-regulated glucose transporter GLUT4 [138]. IRAP contains three domains [136, 139–141]: an intracellular region that is involved in intracellular localization and redistribution, a hydrophobic transmembrane segment, and an extracellular region that contains the catalytic site. The M1 family shares a consensus His-Glu-Xaa-Xaa-His-(Xaa)_18_-Glu zinc-binding motif that is essential for enzymatic activity, and a Gly-Xaa-Met-Glu-Asn (Xaa = Ala in IRAP) exopeptidase motif. The zinc ion is coordinated to the two His residues, the second Glu residue, and a water molecule, which is anticipated to be activated by the other Glu residue during the hydrolytic step [142–144]. Mutational analyses have shown that the Gly-Ala-Met-Glu-Asn motif is important in the recognition of the N-terminal of both substrates and competitive inhibitors [139, 145, 146]. An important and characteristic property of the aminopeptidase is its ability to cleave the N-terminal amino acid residue from several bioactive peptides *in vitro*, including Met-enkephalin and Leu-enkephalin, dynorphin A, neurokinin A, cholecystokinin-8, somatostatin, oxytocin, and vasopressin [135, 147, 148].

Several hypotheses have been presented to explain the ability of Ang IV and its analogues to enhance cognitive functions [25, 149, 150]. One hypothesis concerns substrates such as vasopressin, somatostatin, and cholecystokinin, whose half-lives are prolonged when IRAP is inhibited by Ang IV, LVV-hemorphin-7 or analogous derivatives [151, 152]. These substrates improve parameters associated with cognition [153], and vasopressin and oxytocin are considered to be the main substrates of IRAP [148, 154]. It was recently shown that somatostatin has an impact on memory processing through its action on the somatostatin receptor subtype 3 [155]. A second hypothesis proposes that IRAP acts as a classical receptor that transfers information across the cell membrane after receptor binding, while a third is that the Ang IV analogues prolong the localisation of IRAP and GLUT4 at the cell surface, and thereby modulate the uptake of glucose into neurons and other cells [112, 150, 156, 157]. Furthermore, it has been proposed that the metallopeptidases in the same family as IRAP, such as aminopeptidase N (AP-N, EC 3.4.11.2), are targets [158]. Both Ang IV and LVV-hemorphin-7 inhibit AP-N activity [159]. Alternative macromolecular targets for Ang IV have been proposed, such as c-Met, a tyrosine kinase receptor that binds hepatocyte growth factor (HGF) and that is associated with memory and learning consolidation [160, 161]. Norleual (Figure 6) inhibits HGF-mediated effects at picomolar concentrations and blocks [^125^I]HGF binding to c-Met.

**Figure 8:**
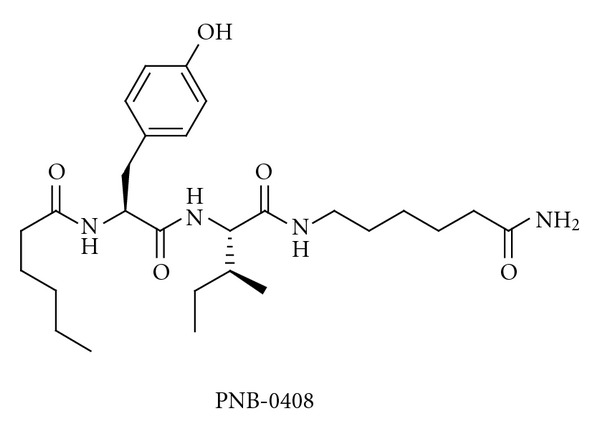
The structure of the compound PNB-0408, which crosses the blood-brain barrier.

The receptor or receptors that are involved in the positive identity identities of the effects of Ang IV and its analogues is still not clear, but both IRAP and c-Met are probably involved [114]. The availability of IRAP/Ang IV receptor ligands that are able to penetrate into the brain is important in order to obtain mechanistic insights. Wright and Harding have synthesised PNB-0408 (N-hexanoyl-Tyr-Ile-N′-(5-carbamoylpentyl)amide, Figure 8) and have shown that it crosses the blood-brain barrier and enhances cognitive activity [133, 162, 163]. This compound should be a very useful research tool. The activity profiles of this modified tripeptide and related analogues have now been established in several models of dementia [133].

Wright and Harding, and their group in USA, have been pioneers in identifying compounds that bind strongly to the Ang IV receptor, as are also Kobori et al. in Japan. Several other research groups have devoted considerable efforts in recent years to developing small molecules that interact with the Ang IV receptor, for example, in Belgium, Sweden, and (in particular) in Australia. These latter three groups have focussed on making efficient inhibitors of IRAP, and they have used slightly different and complementary approaches. The major objectives have been to identify powerful, selective inhibitors that are metabolically stable, and that resist, in particular, degradation by IRAP itself and related peptidases. To make inhibitors with high bioavailability in brain after oral administration is a tremendous challenge.

Many of the ligands previously identified as high-affinity binders were found to inhibit IRAP [111, 164]. Metal chelators (such as EDTA and phenanthroline) had previously been routinely used in experiments that measured binding affinity. It now became important to determine the ability of ligands to inhibit the hydrolysis of synthetic substrates in the absence of metal chelators. Ligands had different potencies and frequently different rank orders in the IRAP assay in the absence of chelators. It was suggested that the differences were a result of the absence of zinc in the active site when metal chelators were present [159, 164–166]. Thus, it became obvious that chelators must be omitted to obtain physiologically relevant results [167]. Furthermore, the results illustrated also the importance of synthesising inhibitors that are metabolically stable and are not substrates of IRAP.

**Figure 9:**
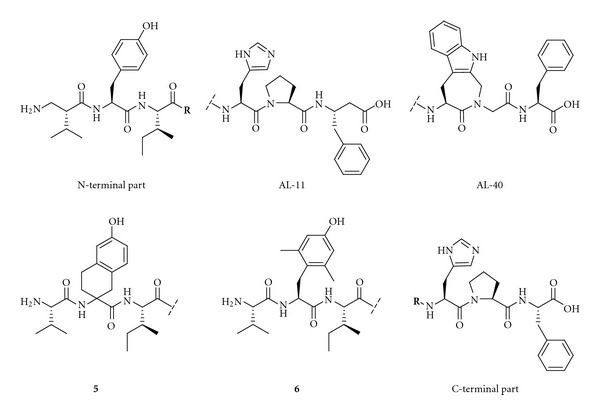
Examples of recently identified IRAP inhibitors.

Lukaszuk et al. in Belgium have recently performed a **β**-homoamino acid scan of Ang IV [168]. Replacement of Val^1^ by (R)-*β* ^2^hVal and replacement of Phe^6^ by *β* ^3^hPhe led to a metabolically stable and potent IRAP inhibitor, AL-11 (Figure 9). This has a high selectivity for IRAP over AP-N and the AT1 receptor. It has been reported that Ang IV affects blood pressure by a process that is mediated by the AT1 receptor [169]. The His^4^ and Pro^5^ residues were subsequently replaced by other, conformationally constrained, residues [170]. Incorporating (R)-*β* ^2^hVal^1^ and Aia^4^-Gly^5^ gave a compound that is highly selective and stable and has a high inhibitory effect (AL-40, Figure 9) [168, 170]. Ang IV itself is only a weak inhibitor of the catalytic activity of IRAP, as has been shown by experiments with the metabolically stable tritiated Ang IV analogue [^3^H]AL-11, in combination with the selective AP-N inhibitor 7B which is a phosphinic transition-state analogue [171] and in the absence of metal chelators. Adding metal chelators creates the apoform of IRAP [167, 172], and it is important to note that the active form and apoforms of IRAP react differently [173]. Hence, much of the previous results refer to binding to the apoenzyme rather than to the catalytically active enzyme [122–124, 174]. Ascher et al. have recently discussed the regulation of the peptidase activity of IRAP in detail [175].

The Belgian group also performed an extensive study that examined the roles of Tyr^2^, Pro^5^, and Phe^6^ in Ang IV by introducing conformational constraints at the different amino acid residues. The study confirmed that conformational constraints are important in obtaining selectivity. Replacing Tyr^2^ by any one of several conformationally constrained residues impairs the activity of the peptide, while modifications at the C-terminal are more acceptable. Analogues **5** and **6** (Figure 9), for example, inhibit IRAP to a very low degree. This suggests that the orientation of the Tyr^2^ side chain is critical for activity [176].

**Figure 10:**
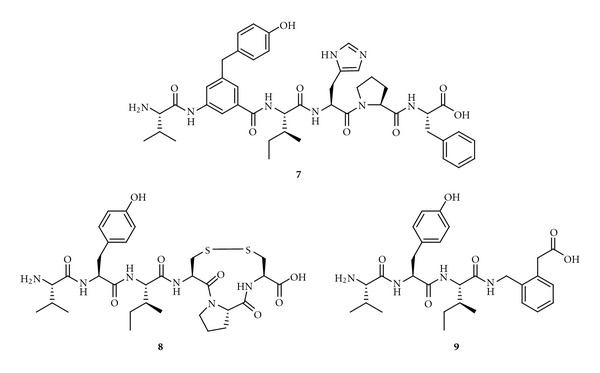
The structures of the Ang IV peptidomimetics **7**–**9**.

Our approach in Sweden to obtain active inhibitors of IRAP has been based on an ambition to determine various bioactive conformations by introducing local steric constraints. We have also worked to create various secondary structures by side chain cyclizations. Incorporation of a 4-hydroxydiphenylmethane scaffold as a substitute for Tyr^2^ as part of the attempts to create steric constraint, as in Compound **7** (Figure 10), is deleterious for activity. This result again suggested that position two is susceptible to structural manipulation [177]. A series of macrocyclizations was performed in order to obtain improved inhibitors and to better understand how Ang IV binds to IRAP [174, 178–180]. Cyclization of [Cys^4^, Cys^6^]Ang IV to form an 11-membered ring resulted in a compound (**8**, *K*_*i*_ = 26 nM, Figure 10) that is more potent than Ang IV (*K*_*i*_ = 62 nM) as an IRAP inhibitor. Previous conformational analyses of Ang II suggested that such a macrocyclic system would tend to adopt an inverse *γ*-turn [181, 182], and the replacement of His-Pro-Phe by a 2-(aminomethyl)phenylacetic acid moiety designed to mimic an inverse *γ*-turn resulted in compound **9** (Figure 10). This compound has a *K*_*i*_ value of 44 nM and a much simpler structure than previous candidates [171].

**Figure 11:**
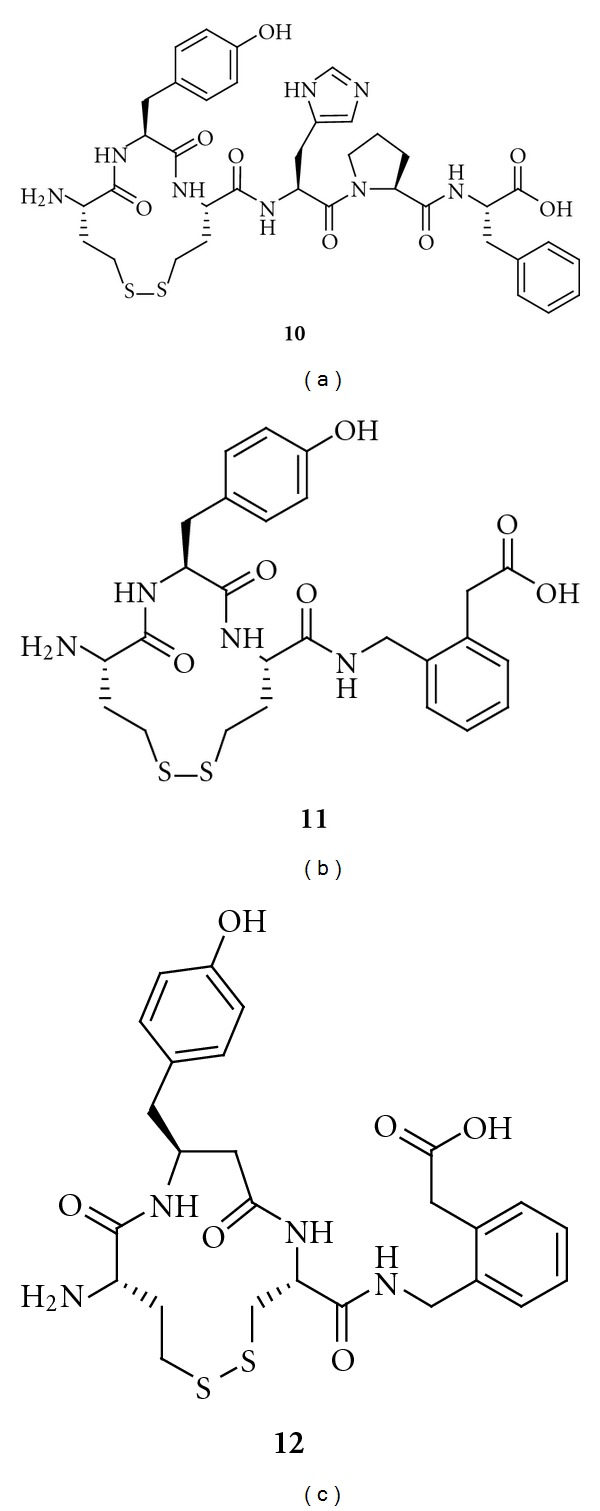
The structures of three IRAP inhibitors from a series of disulfide-cyclized Ang IV peptidomimetics. The ability to inhibit IRAP increases from **10** to **12**. Both **11** and **12** have 2000-fold selectivity for IRAP over AP-N.

Cyclization of [Cys^1^, Cys^3^]Ang IV gives an inactive inhibitor with an 11-membered ring system, while a compound with fair inhibitory potency and a 13-membered macrocycle is obtained using [Hcy^1^, Hcy^3^]Ang IV (**10**, *K*_*i*_ = 303 nM, Figure 11). The hybrid of **9** and **10** is a potent inhibitor (**11**, *K*_*i*_ = 23 nM, Figure 11), and, importantly, selective for IRAP over AP-N. Thus, it appears that that Ang IV adopts a *γ*-turn at the C-terminal when binding to IRAP, while an open, less well-defined turn conformation is present at the N-terminal. Further structural optimisation produced compound **12** (Figure 11), which has a *β* ^3^hTyr residue in a 13-membered macrocycle. Its *K*_*i*_ value is 3.3 nM. This compound is 20 times more potent than Ang IV and exhibit a 2000-fold selectivity for IRAP over AP-N [180]. Removal of the carboxyl group at the C-terminal gives less efficient inhibitors of IRAP. Hence, macrocyclizations by oxidative disulfide formations can provide very potent IRAP inhibitors.

**Figure 12:**
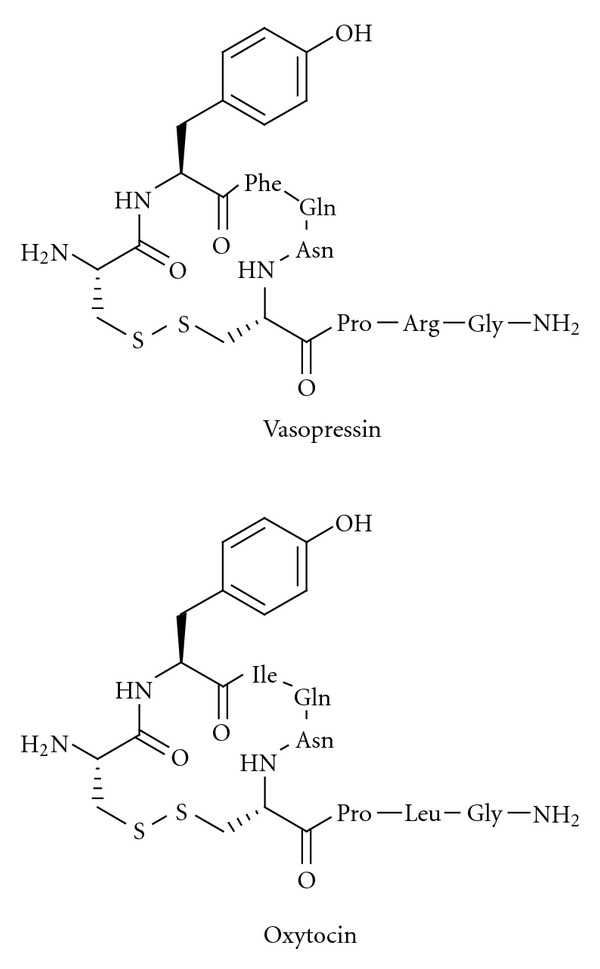
The macrocyclic disulfides vasopressin and oxytocin are substrates of IRAP.

Macrocyclizations, obtained by applying the metathesis reaction, are very attractive in efforts to make potent compounds with high oral bioavailability [183, 184]. This observation and the knowledge that compounds such as vasopressin and oxytocin (Figure 12) are macrocyclic in the N-terminal part and substrates of IRAP [148] prompted the synthesis of carba analogues. It was believed that these would be efficient IRAP inhibitors that were more metabolically stable.

**Figure 13:**
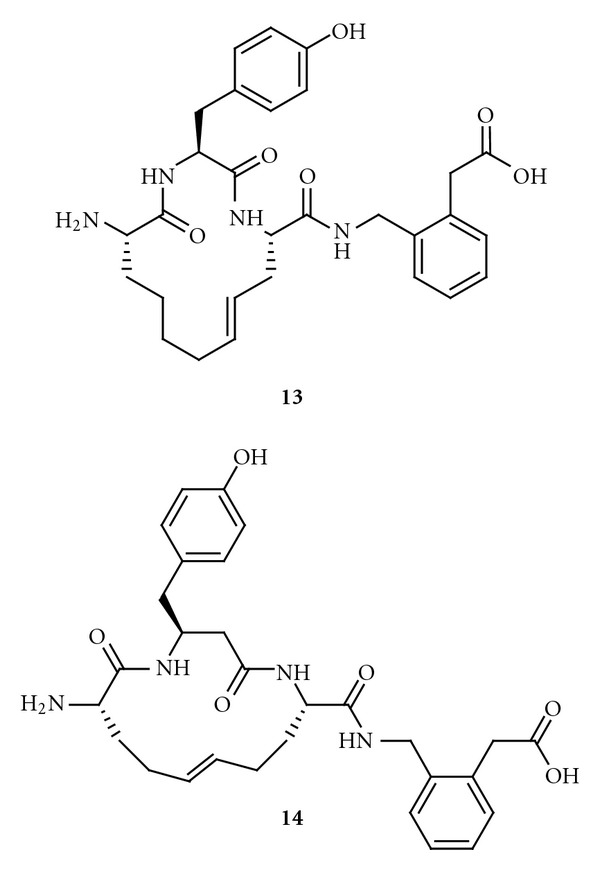
Macrocyclic Ang IV peptidomimetics that strongly inhibit IRAP.

A large series of macrocyclic compounds were prepared, of which the compounds labelled **13** and **14** (Figure 13) have the lowest *K*_*i*_ values, 4.1 nM and 1.8 nM, respectively [179]. These carba analogues are also the most metabolically stable analogues that were synthesized in the programme. In the absence of chelators, the binding affinity of the 14-membered macrocyclic compounds to IRAP is 10 times higher than that of Ang IV. N-Methylation of the peptide bond between residues one and two reduces the activity, suggesting that the amide nitrogen and the N-terminal primary amine nitrogen are coordinated to the zinc atom in the active site of the protease [179]. Incorporation of a methylene group adjacent to the N-terminal amino group, as in AL-11 and AL-40 (Figure 9), and replacement of the C-terminal carboxyl group with bioisosteres seem to be the obvious next steps in efforts to improve the inhibitors. The amide bond between residues one and two, which is present in all of the macrocyclic inhibitors that have been reported, is the major target for IRAP. Thus, exchanging this bond for a proteolytically inert CH_2_NH fragment may improve the properties. Such a CH_2_NH fragment should provide also a strong coordination to zinc.

The Australian group discovered a series of IRAP inhibitors based on a benzopyran system as scaffold. These drug-like compounds should be more metabolically stable than the peptidomimetics previously discussed [113]. While compounds **13** and **14** were produced through various modifications of pre-existing compounds, primarily macrocyclizations of Ang IV itself, inhibitors based on benzopyrans originate from a structurebased design process and virtual screening.

**Figure 14:**
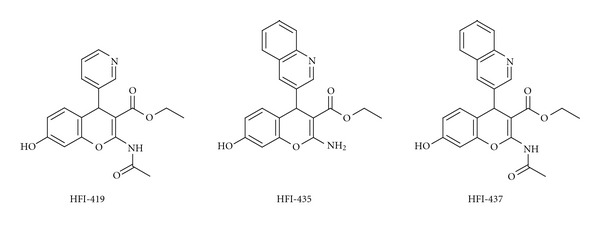
Recently identified potent non-peptidic IRAP inhibitors with a benzopyran scaffold.

The 3D structure of IRAP is not known. Thunnissen et al. therefore used the structurally related human leukotriene A_4_ hydrolase [185], which also belongs to the M1 aminopeptidase family, in an *in silico* screening aimed at identifying potential inhibitors [186]. The process involved the *in silico* screening of 1.5 million commercially available compounds against a model structure homologous to IRAP, identification of hits, biological evaluation of hits, and optimisation of structures. Several potent drug-like benzopyrans were identified as IRAP inhibitors. Among those, the racemic pyridine derivative HFI-419, and the quinoline derivatives HFI-435 and HFI-437 (Figure 14), are selective for IRAP, with *K*_*i*_ values of 420, 360, and 20 nM, respectively. The compounds bind with low affinity to structurally related enzymes, such as AP-N and leukotriene A_4_ hydrolase itself, despite the latter having been used in the homology modelling [186]. Computational docking suggests that the *S*-isomer is the preferred binding mode in all examples, and two alternate binding conformations for these structurally analogous inhibitors have been proposed [187].

**Figure 15:**
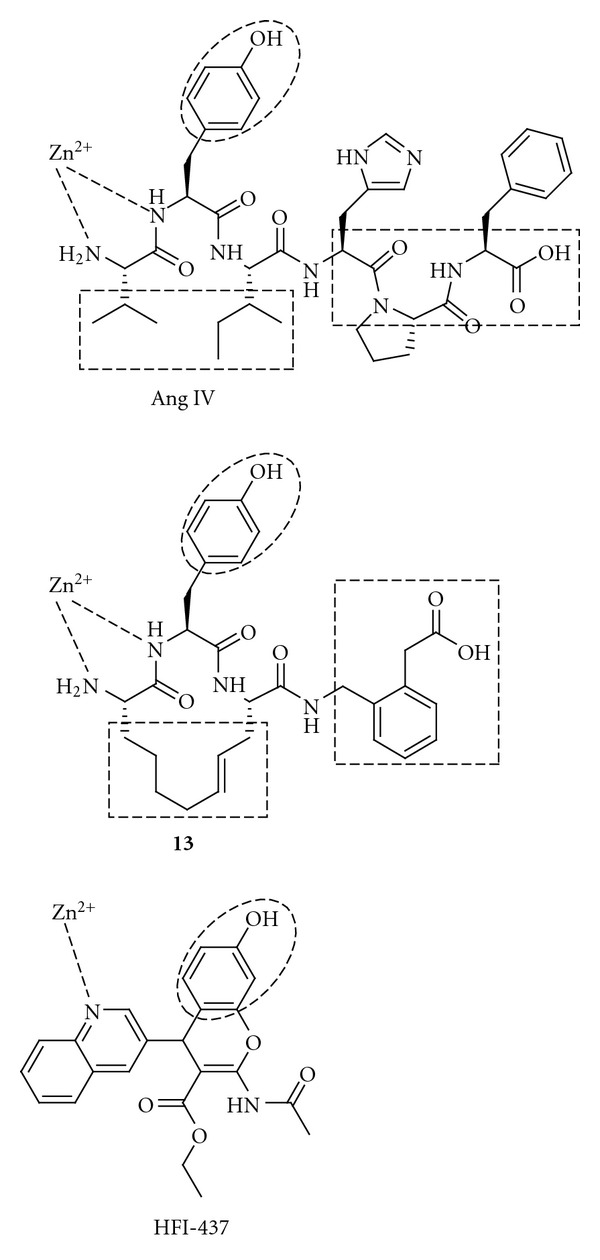
Ang IV, a peptidomimetic (**13**) and a non-peptidic (HFI-437) IRAP inhibitor. The rectangles and circles show proposed recognition elements important for interaction with IRAP.

The quinoline compounds HFI-435 and HFI-437 cannot adopt the same binding mode as the pyridinyl compound HFI-419 for steric reasons, but HFI-435 and HFI-437 seem to adopt binding modes that allow a stronger interaction with the zinc ion through coordination with the nitrogen atom in the quinoline heterocycle. It has been predicted that the quinoline compounds are more active than the pyridinyl compounds, partly due to a more favourable coordination to the zinc ion in IRAP. In addition, computational docking experiments have suggested that Phe^544^ of IRAP provides an important hydrophobic packing point at one side of the active site [187]. No comparative modelling has been carried out, but it is tempting to suggest that the N-terminal of Ang IV, the macrocyclic compound **13** and the quinoline HIF-437 bind to IRAP as shown in Figure 15. The amide nitrogen (or the amide oxygen) and/or the N-terminal nitrogen atom may interact with the zinc atom.

Albiston et al. demonstrated that *i.c.v*. administration of HFI-419 enhances memory in two memory paradigms. The performance of rats in a task involving spontaneous alternation of spatial working memory after administration of compound HFI-419 [186] was similar to the performance after administration of Ang IV and LVV-hemorphin-7 [14]. Results from *in vivo* experiments with this drug-like class of compounds strongly support the strategy of using IRAP as a target for cognitive enhancers. The benzopyran class of IRAP inhibitors provides promising leads for further development.

## 6. Conclusions

Experimental data from various animal models demonstrate that inhibitors of IRAP facilitate memory. The explanations to the beneficial effects on a biochemical level are not clear and many alternative hypotheses have been proposed. Further studies to elucidate the mechanism of action are needed. In this context, metabolically stable IRAP inhibitors, able to cross the blood-brain barrier and with fair bioavailability in brain should serve as important research tools. Such inhibitors could also provide new chemical entities and enhancers of cognition for future treatments of the memory loss associated with ageing and AD. All data with IRAP inhibitors available today were achieved after i.c.v. administration and new inhibitors with oral bioavailability are highly desirable. There are in brief two different approaches addressing the discovery of such inhibitors. One starts from Ang IV itself. After subsequent truncations and macrocyclizations, stabilizing favourable conformations, potent inhibitors could be obtained. However, these are still peptidic in character. The other approach originates from a virtual screening and the utility of a homology model of IRAP. A series of drug-like small inhibitors with high potential for further optimization were identified by this process. The major challenge remaining for these inhibitors is to enable them to cross the blood-brain barrier.
